# Thermal Conductivity of Sand-Lime Products Modified with Foam Glass Granulate

**DOI:** 10.3390/ma14195678

**Published:** 2021-09-29

**Authors:** Iga Jasińska, Ryszard Dachowski, Monika Jaworska-Wędzińska

**Affiliations:** 1 Faculty of Mechanical Engineering, Kazimierz Pulaski University of Technology and Humanities in Radom, Ul. Malczewskiego 29, 26-600 Radom, Poland; m.jaworska@uthrad.pl; 2 Faculty of Civil Engineering and Architecture, Kielce University of Technology, Al. Millennium of the Polish State 7, 25-314 Kielce, Poland; tobrd@tu.kielce.pl

**Keywords:** thermal conductivity, sand-lime products, foam glass granulate, modified, recycled glass

## Abstract

Waste glass constitutes a significant part of general waste worldwide. Unfortunately, only a small percentage is recycled. It is, therefore, quite important that it can be applied in the production of construction materials. The main aim of this article is to determine the thermal conductivity of the products modified with granulated foam glass (GFG) (recycled product) of the 0.25–0.5 mm fraction, as well as to indicate dependence of the change in volume density of samples caused by the use of GFG and the change of the thermal conductivity coefficient compared to reference samples. For the purpose of this research, various parameters were examined i.a. volume density, water absorption, determination of the pore size distribution by mercury porosimetry and determination of the heat conduction coefficient with the use of a plate apparatus. The test results were developed on the basis of a mathematical model that determined the influence of the filler on the functional properties of the product. The research has shown that the use of GFG in the sand-lime products will contribute to lowering their thermal conductivity by more than 50% compared to traditional products.

## 1. Introduction

Waste glass constitutes a significant part of general waste, and its collection becomes a problem for almost every country. Although large amounts of waste glass are generated every year worldwide, only a small percentage is recycled. Despite numerous attempts and initiatives, the management of glass cullet that is formed outside the glass production process, i.e., recycled cullet, did not bring any satisfactory results [1,2]. Environmental protection measures aimed at the sustainable use of resources as well as the problem of solid waste management provide a strong encouragement for their reuse, which is in line with the idea of sustainable economic development.

Landfills are filled with large quantities of glass residues and their amount is still growing [3]. In 2018, the total amount of waste generated in the EU by all economic activities and households reached 2277 million tonnes (including glass waste). In the same year, 54.2% of waste was subject to recovery processes, 38.1% of which was recycled. The remaining 45.8% was disposed in landfills. Taking this into account, there is no surprise that one of the goals of the EU is to reduce the amount of waste generated as well as to promote it as a resource in order to achieve higher recycling rates and safe disposal of waste [4]. It is therefore important to notice that glass waste has great potential to be used as a raw material in the construction industry. Scientific research shows that its use in building materials leads to improvement of their physical characteristics. The use of waste glass in building materials may minimize the costs of production and also reduce the burden on landfills [5]. Given the above interest in using waste as alternative aggregates, its role should further explored in the area of building materials.

Waste glass can be used as a substitute to commonly known construction products. The products with new physical and mechanical characteristics can be used both in the current and new forms of these products. Thus, the role of waste glass management in the construction process is often undertaken by many researchers both in terms of their use in road [6,7] and general construction [8].

The use of waste glass is widely reflected in tests carried out on cement composites, i.e., mortars and concrete. It is mainly used as a substitute for fine aggregate in the form of glass sand, or coarse aggregate—as glass cullet [9,10,11,12].

Małek, Łasica et al. [13] indicate that with the increase of glass sand content, the mortar density decreased. This effect was, however, caused by the lower specific gravity of glass cullet than granite sand and the use of green glass aggregate with increased Mohs hardness.

Sikora, Horszczaruk et al. [14] analyzed the mechanical and thermal properties of cement mortars, where fine aggregate was replaced with soda-lime aggregate (waste glass). Also, 0, 1 and 3 wt.% of nanosilica was added to the cement mortar. The experiment proved that the presence of fine soda-lime aggregate significantly lowered the thermal conductivity of cement mortars. The study showed that fine aggregate (WG) can replace natural fine sand without the consequences of mechanical deterioration and with a significant improvement of the thermal characteristics of cement mortars. In addition, adding nanosilica (especially in higher concentrations—3%) leads to further decrease in thermal conductivity and improvement in compressive strength at the same time.

Bastanci [15], on the other hand, stated that for the concrete made of natural aggregate and marble dust (replacement of Portland cement) and mixtures of Portland cement in which 20% of the natural sand was replaced by sand glass, compressive strength was reduced by up to 19%. However, in the same case other parameters have improved significantly, including sound insulation, thermal conductivity, and water resistance.

Using waste in the construction industry cannot be limited only to cement-based composites. Sustainable development has to be also taken into consideration. For the purpose of this publication, it should be noted that there are many possibilities of using waste as fillers or substitutes for the mixture in the area of sand-lime products. Such modifications are not so common, but they constitute an important reference in the development of research on construction materials. These products differ from the above-mentioned cement composites mainly in their composition—traditional products are made of three basic components—quartz sand, lime and water—and the manufacturing process, as the curing takes place in an autoclave (under high temperature and pressure conditions—203 °C and 1.6 MPa, respectively). The current research on changing the composition of the sand-lime products is mainly aimed at improving compressive strength or their acoustic properties. The fillers used in the mass of such products included components of both natural [16,17] and chemical origin [18,19]. However, recycled products [20,21], polypropylene mesh [22], other production wastes and leachate from landfills [23,24] were also taken into account. The newest research on the sand-lime products is also based on their modification with waste glass in the form of glass sand and glass cullet [25,26,27]. Similarly to cement composites, these studies have shown that the increase in the amount of glass sand contributes to the higher compressive strength of these products, while the bulk density and water absorption of these samples is lower [25,26,28]. These tests completely described the changes in the microstructure resulting from the modifications to the composition of the initial mixture.

One of the advantages that characterizes traditional sand-lime products is their compressive strength of 15–20 MPa. Their relatively high values of thermal conductivity are considered a disadvantage of these products. This problem is even more important as the applicable regulations rigorously tighten the requirements for thermal insulation and energy consumption of buildings. Consequently, materials with a low thermal conductivity coefficient should be sought both in terms of construction materials and insulation. This kind of material will also have a positive impact on the weight. One of the methods to produce this kind of products is through the use of lightweight, porous fillers in the sand-lime mass.

Particular attention has been paid to white foamed glass in the form of granules as a product of glass cullet recycling. Its use as a waste material for the production of construction materials can significantly limit the costs related to environmental damage, as well as promoting sustainable use of natural resources.

The use of granulated foam glass as well as expanded glass is reflected in the modification of cement composites [29,30,31].

Limbachiya et al. [32] examined the possibility of using granulated foam glass with a coarse and fine fraction as a volumetric substitute for natural aggregate in the production of concrete. The results of the compressive strength tests showed that the addition of up to 30% of coarse-grained or 5% of fine granulated foam glass has a substantially negligible effect on the compressive strength of the concrete sample. Regardless of the amount of granulated foam glass, the compressive strength after seven days increased to the range of 73–87% and was comparable to the strength of concrete based on natural aggregate.

Vangonov et al. [33] explored the use of expanded glass granules in high and low density foam concrete. Based on the results obtained, it was presented that the addition of light foamed glass grains reduces the compressive strength of high-strength foam concrete and does not affect the strength of low-foam concrete. The advantage of the addition of foamed glass is a reduction in density (especially in case of high-density foam concrete) and a significant reduction in surface water absorption in relation to the samples that do not contain a light additive). Other studies considering the use of granulated foam glass have shown that its presence in the sample weight contributes to an increase both in sound absorption [34] and in thermal insulation [35].

On the other hand, the overall aim of the work by Kurpińska and Ferenc [36] was to investigate the properties of various types of lightweight concretes containing expanded granulated glass. Although the use of the light aggregate resulted in a reduction of the mechanical properties of it, such as compressive strength or modulus of elasticity, it also revealed certain advantages and improved the properties of concrete, i.e., thermal and acoustic insulation.

However, the current research does not provide any information on thermal insulation properties of the sand-lime products mixed with granulated foam glass. It can be estimated that a light filler in the mass of a sand-lime product may improve its thermal insulation characteristics, which eventually may result in the use of these products in lightweight structures as an insulation material. Therefore, the main goal of this article is to determine the thermal conductivity of the products modified with a lightweight filler i.e., granulated foam glass, as a recycled product with a fraction of 0.25–0.5 mm. The paper also attempts to indicate the dependence of changes in the volume density of samples caused by the use of this lightweight filler, and changes in the thermal conductivity in relation to reference samples made on the basis of a traditional sand-lime mixture.

With regard to sand-lime products, there have been a number of studies on the possibility of using granulated foam glass (GFG), but they are related mainly to its microstructure and functional properties [37,38]. This article complements the research published in the article [39] on changes in the microstructure of products modified with foamed glass granules in the dimensions of 0.25–0.5 mm. Previous research and presented results [39] have shown that with a decrease in bulk density the compressive strength of products modified with expanded glass granules decreases. That is related both to the properties of the filler (GFG) and to the changes in the microstructure of the samples examined.

## 2. Materials and Methods

The samples used for this research included the 40 × 130 × 160 mm blocks, to test the heat conduction coefficient, and the 40 × 40 × 160 mm bars. They were selected in order to examine specific functional properties such as volume density and water absorption. The starting mass and at the same time the mass of the traditional (standard) product was a silicate mixture (lime-sand) consisting of sand made up of silica (92%) and hydrated lime (8%). The molar ratio of the starting mass is CaO/SiO_2_ (C/S) = 0.09. In order to obtain the sand-lime products modified with granulated foam glass, a mass consisting of 70–95% of a sand-lime mixture and the appropriate amount of filler was prepared. In every case, the total mass of the mixture and the filler was equal to 100%. It was necessary to prepare the bars to determine the volumetric density and water absorption. The granulated foam glass ranged between 5–30%, with a measuring step every 5%. The blocks were also made to determine the thermal conductivity coefficient. In case of these blocks, the filler was used in the amount of 5, 10, 20 and 30%, respectively. This mixture was filled up with 6% of water of the mass obtained. Subsequently, the mass, similarly to the mass for traditional products, was formed into cuboidal elements and pressed with a force corresponding to 20 MPa and placed into an autoclave for 8 h at a temperature of 203 °C (traditional autoclaving process).

Before testing, the samples were air-dried for 14 days at a temperature of 20 °C and relative humidity of ≤65%, following the applicable standard [40]. Porosity was tested using the mercury porosimetry method. In this case, the sample was taken from the bars with 5, 10 and 30% of the filler content and with volume density, which was the closest to the average result obtained from the volume density tests.

### 2.1. Fine Sand

The raw material constituting the aggregate was natural quartz sand. The results of the granulometric analysis were based on the PN-EN ISO 14688-2: 2006 [41]. The sand used in the research consists of a sand fraction (grain diameters from 0.063 ÷ 1.0 mm) and is classified as medium sand (MSa).

### 2.2. Lime

The samples were made of ground burnt lime, the main component of which is calcium oxide CaO (CL 90-Q, R5, P1). It is obtained by evenly firing limestone at a temperature of 900–1300 °C, and then grinding in a mill. The chemical composition of lime obtained on the basis of the Quality Control Certificate is presented in Table 1.

### 2.3. Characteristics of the Filler 

**Figure 1:**
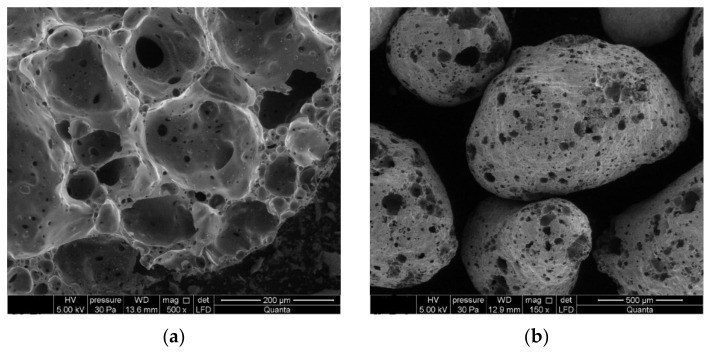
Expanded granulated foam glass—SEM pictures (x250): (**a**) granulate surface, (**b**) cross-section of the granulate.

The filler used in the sand-lime mass was granulated foam glass in the dimensions of 0.25–0.5 mm. The morphological features of the granules were examined using scanning electron microscopy (SEM). A characteristic feature of this product is its porous and cellular structure. As presented in Figure 1a, the grain surface is rough with visible sparse and small pores. Along the grain circumference, there are small open pores visible in the structure (Figure 1b). The core of the granules consists of large pores. Because of the porous structure, the granulated foam glass is characterized by a low thermal conductivity coefficient of 0.06–0.07 W/(m·K). The characteristic of the product shows that the full water saturation of the granulated foam glass does not exceed 10% of the total volume of the material. Additionally, the structure of open pores of various dimensions contributes to a very good sound absorption, which can be then used to regulate acoustics in various rooms.

### 2.4. Bulk Density Test

The bulk density test was carried out in accordance with the standard [42]. For the purpose of this study, the samples used had dimensions of 40 × 40 × 160 mm. The measured dimensions (length, height and width) allowed determination of the volume of the sample. The stamps were then dried in a circulation dryer at 105 °C to achieve a stable weight. Based on the results obtained, the values of volumetric density for each element were calculated in accordance with the guidelines of the particular standard [42]. 

In each case, the study of the bulk density was carried out five times, i.e., on six samples of the same composition. The arithmetic mean of the calculated values was assumed as the result of the determination. The dependence of the volumetric density on the amount of filler in the sample is presented in the article on selected functional properties and microstructure of sand-lime samples modified with granulated foam glass [39]. In this publication, the results obtained in terms of bulk density were used to determine the relationship between the bulk density and water absorption of samples of the same composition.

### 2.5. Water Absorption Test

The water absorption test was carried out in accordance with the guidelines included in applicable standards [43,44]. The prepared samples were dried at 105 °C until their stable weight was obtained, followed by their cooling. Afterwards, the cooled sample was gradually covered with water at room temperature, followed by its immersion in water to half of their height at first, and complete immersion after 2 h. 48 h later, the samples were removed, excessive water was wiped off from the surface and the samples were weighed. The compressive strength test was repeated five times in each case. Based on the values obtained, the absorption parameters for each element were calculated in accordance with the guidelines contained in the aforementioned standards. The arithmetic mean of the six calculation results on samples of identical composition was assumed as the result of the determination.

### 2.6. Determination of the Pore Size Distribution by Mercury Porosimetry 

Mercury porosimetry is considered to be the most universal and the most commonly used method of porosity analysis of construction materials. This examination is based on measuring the volume of mercury penetrating into the pores at a specific equilibrium pressure. Thanks to this method it is possible to determine their volume as a function of diameter. This method allows for the identification of open pores forming a network of connections [45].

Mercury porosimetry aims to carry out a qualitative assessment—through the analysis of the pore space, and quantitative assessment understood as the determination of physical parameters of this space [46,47].

The laboratory test was carried out using a PoreMaster60 mercury porosimeter by Quantachrome on cuboidal samples with dimensions of 10 × 10 × 20 mm. In this apparatus, thanks to the computer-based mercury injection process from the pressure lower than the ambient pressure to the value of 413.4 MPa, the liquid penetrates the pores. It was, therefore, possible to determine the basic parameters characterizing the pore space of the samples tested.

In this study, the pore size distribution, introduced by the International Union of Pure and Applied Chemistry (IUPAC) [48,49] was used to evaluate the pore size distribution. The conducted research allowed for the analysis of porosity in the pore size ranged from 3 nm to 200 µm, which means that the pore size distribution covers the range of mesopores and macropores. Graphical representation of the results are plots of cumulative curves of pore volume distribution in relation to their pore diameter.

### 2.7. Determination of the Thermal Conductivity Coefficient

Thermal properties of construction materials are characterized by the thermal conductivity coefficient (λ). It is an important parameter for highly porous materials that are used as thermal insulation. Its value depends primarily on the total porosity. Other features influencing its value are the size and shape of pores, sample humidity, temperature, and chemical and phase composition, e.g., the content of the amorphous phase [49,50].

Tests performed to determine the value of the thermal conductivity coefficient for the traditional product and modified sand-lime products were undertaken on the samples in the dimensions of 160 × 130 × 40 mm. The test was undertaken using a plate apparatus with heat flux density sensors (typ FQ90xxx) according to ISO [51]. It was a single-sample system with the measurement area of 100 × 100 mm. During the measurement, the values of signals from the two types of sensors were controlled, thanks to which thermal parameters at the input and output from the sample were obtained, i.e., heat flux density and surface temperature of the plates, respectively.

The surfaces of the blocks were levelled by grinding to ensure that the surface of the examined material was as close as possible to the measuring plate, while maintaining the parallelism of the face surfaces (160 × 130 mm). The prepared blocks were carefully measured and weighed in order to determine their real volume density. Completion of all these actions allowed us to proceed with the appropriate thermal conductivity tests. Each time, the study was repeated twice. The data obtained are presented in a tabular and a graphic form with description.

## 3. Results

### 3.1. Dependence of Water Absorption on Volume Density

**Figure 2:**
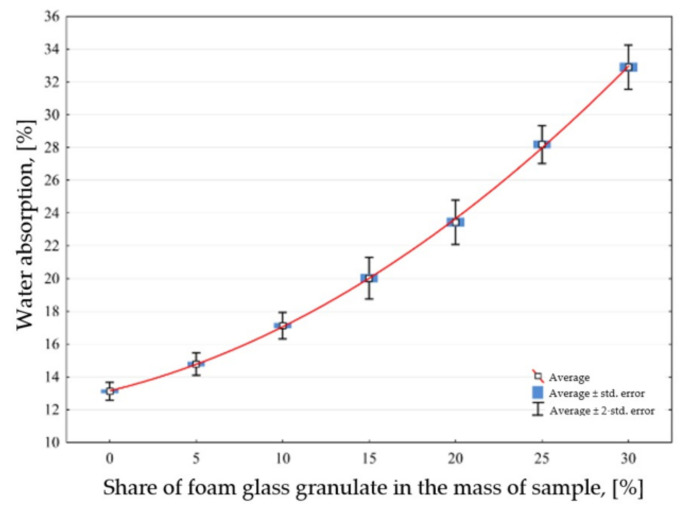
Effect of GFG fraction 0.25–0.5 mm weight of the sample to the water absorption of the final product.

The results of the water absorption tests for the basic sand-lime samples and samples modified with granulated foam glass of 0.25–0.5 mm are presented in the diagram (Figure 2). The results allowed the determination of the shape and the form of the curve describing the relationship between the bulk density and the use of granulated foam glass with a fraction of 0.25–0.5 mm in relation to the sample weight.

The test results show that with the increase of the filler in the sample mass, the water absorption of the samples increases. The average value of the water absorption of the tested reference samples is 12.13% (in the absence of filler), while 30% of the filler in the sample mass contributed to the increase in the water absorption to the average value of 32.90%. This means that these samples absorb almost 2.5 times more water than the reference samples. This has been found to be related to the increase in the specific surface area of the samples as a result of the use of a lightweight filler with a porous structure.

Similar studies were carried out for high and low density foam concrete [33] but the results indicate that the absorption of the obtained products was reduced. This may be due to the technological differences of the samples, which, according to [33], cured at the ambient temperature. It can be assumed that the increased water absorption may be a result of the lime hydration reaction and its impact on the surface of the granulate used during autoclaving. However, this hypothesis should be further explored in additional research.

For the obtained test results, the shape and form of the curve describing the relation between water absorption and the fraction of foam glass of 0.25–0.5 mm in the product weight was determined. The curve represents the value of the water absorption that is dependent on the proportion of GFG filler added in the mass of the sample, which can be described by the formula:

(1) $$A_{1P}=13.15+0.26\;\cdot \;x_{1P}+0.014\;\cdot \;x_{1P}^{2}\pm 0.52$$

The indication in the formula: A_1P_—water absorption of the sand-lime product modified with GFG, size 0.25–0.5 mm, x_1P_—share of GFG in the modified lime-sand product.

**Figure 3:**
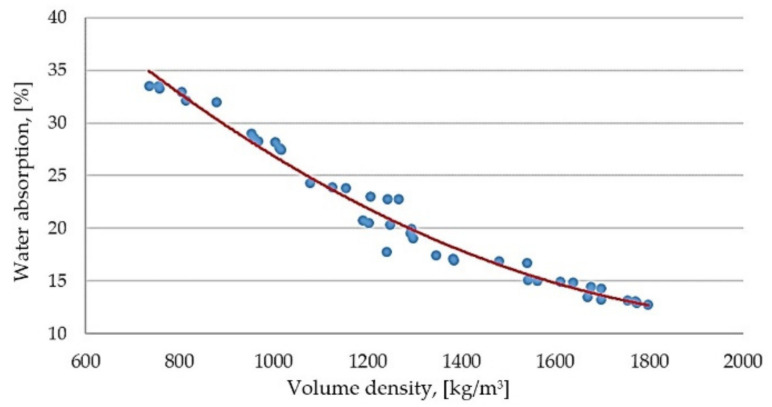
Dependence of water absorption on the volume density of modified samples with the fraction GFG of 0.25–0.5 mm.

Based on the results obtained, it was also possible to determine the shape and form of the curve describing the relationship between water absorption and the volume density of the modified GFG samples (Figure 3).

The water absorption value is closely related to the volume density—water absorption decreases along with the increase in the volume density of the samples. The curve representing the dependence of water absorption on the volumetric density of the GFG-modified sand-lime products can be described by the formula:

(2) $$A_{1P}=66.03+0.51\;\cdot \;\rho_{1P}+1\;\cdot \;10^{-5}\;\cdot \;\rho_{1P}^{2}$$

The indication in the formula: A_1P_—water absorption of the sand-lime product modified with GFG, size 0.25–0.5 mm, ρ_1P_—volume density.

The adopted model explains the variability of the bulk density in 98%.

### 3.2. Pore Size Distribution

The pore size distribution was examined using the mercury porosimetry method on the samples containing 5, 10 and 30% of GFG, named 1P5, 1P10, 1P30, respectively. To compare the results, a characteristic curve for the traditional product obtained (marked as WP) has also been attached.

**Figure 4:**
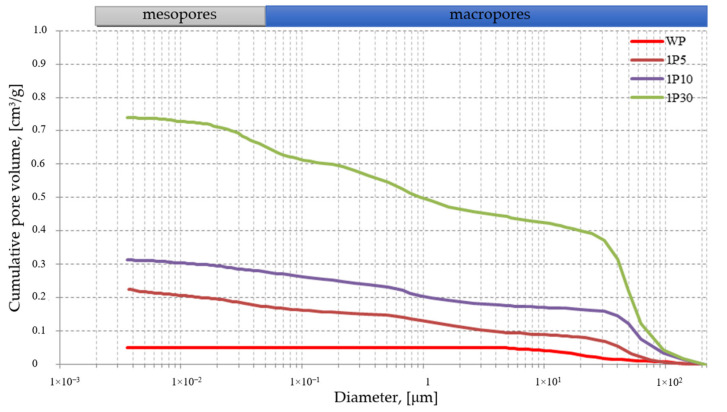
Cumulative pore volume distribution curve for a modified GFG product with a size of 0.25—0.5 mm.

For the purposes of this test, the samples were taken from the bars, the volume density of which was closest to the result of the average density obtained from the measurements of the samples taken. The applied measurement method allowed the visualization of the pore volume distribution depending on their size in the form of a cumulative pore distribution curve (Figure 4). Considering qualitative assessment, it should be noted that the results obtained from the samples taken from the modified GFG products showed the presence of open pores with the dimensions corresponding to mesopores and macropores in the range of measured diameters. Their presence is related to the porous structure of the filler. The largest part relates to pores with a diameter greater than 30 µm for all samples analyzed and in the diameter range of 1.6 ÷ 2.1 · 10^−2^ µm in the case of a sample containing 30% of GFG, as evidenced by the high slope of the obtained distribution curves. The macropores have the predominant part of the volume in each of the samples.

The physical parameters of the pore space obtained on the basis of the tests performed with the mercury porosimetry method are presented in Table 2. These results also constitute a quantitative assessment of the examined pore space.

In the analyzed samples, the average diameter (d_50_) is in the range of 20 ÷ 31 µm. The porosity of the tested materials increases with the increase in the share of 1P filler from 35.49% in the 1P5 sample to 62.31% in the 1P30 sample.

The results of the research show that the effective porosity of the samples increases significantly with the GFG in the mass of the samples and is more than seven times higher in the samples containing 30% of GFG as compared to the reference samples (WP). In each of the analyzed samples, macropores play a significant role in the structure. Referring to the research results presented in the article [41], the structure of the products is well integrated indicating the durability of the products obtained.

### 3.3. Dependence of Thermal Conductivity on Bulk Density

The thermal conductivity of the samples modified with granulated foam glass was tested with a single repeat of the measurement. Samples containing 5, 10, 20 and 30% of GFG with fraction of 0.25–0.5 mm were used for the study, marked in the test results as 1P5, 1P10, 1P20 and 1P30, respectively. With regard to the bars, the bulk density of the tested blocks was determined in accordance with the standard [42]. The obtained and averaged measurement results are presented in Table 3.

**Figure 5:**
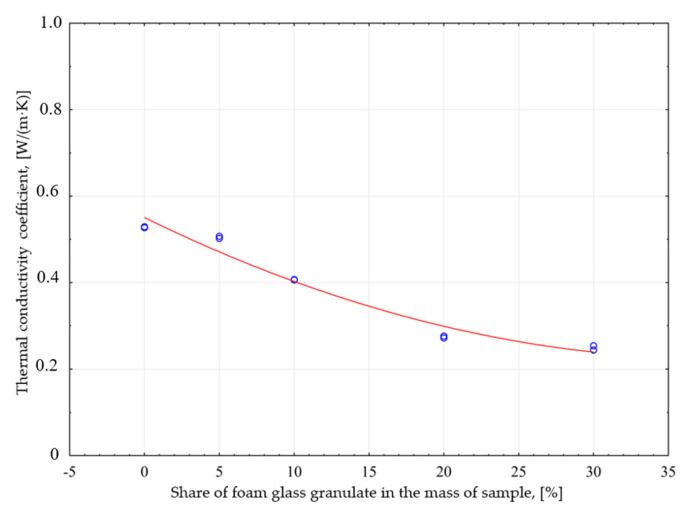
Influence of the GFG content of 0.25–0.5 mm fraction in the sample weight on the thermal conductivity of the tested samples.

Based on studies performed, a curve of thermal conductivity λ of the share GFG by weight of the modified product can be seen (Figure 5).

The test results confirm that with the increased proportion of the filler, and as a result with the increased number of pores in the sample mass, the thermal conductivity coefficient decreases, which is a positive effect of the modification. This significant relationship is presented by the quadratic function described by the formula:

(3) $$\lambda_{1P}=0.55-0.017\;\cdot \;x_{1P}-0.0002\;\cdot \;x_{1P}{}^{2}\pm 0.02614$$

The indication in the formula: λ_1P_—thermal conductivity coefficient of a sand-lime product modified with GFG, size 0.25–0.5 mm, x_1P_—share of GFG in the modified lime-sand product. The adopted model explains the variability of the bulk density in 96%.

**Figure 6:**
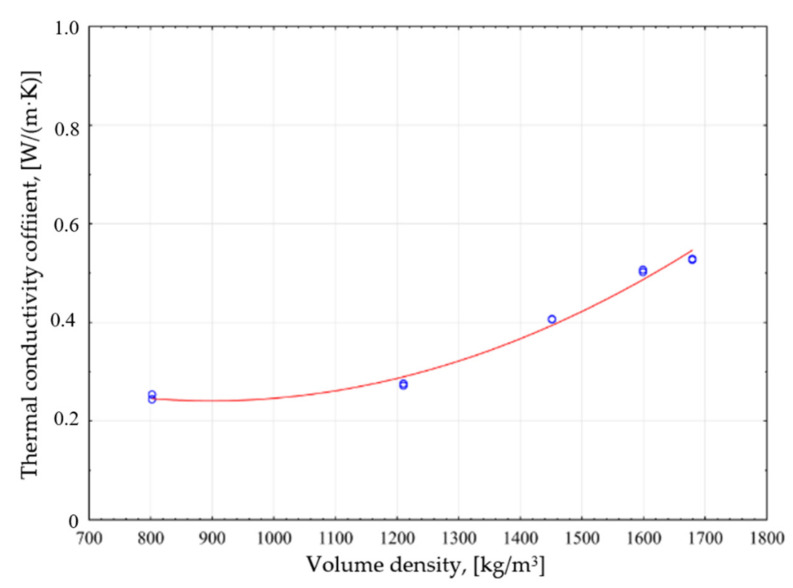
Dependence of thermal conductivity on the volume density of modified GFG samples with a fraction of 0.25–0.5 mm.

It is important to determine the dependence of the bulk density and the thermal conductivity coefficient for the tested samples. The research results obtained allowed for the drawing of the curve of this dependence (Figure 6).

The dependence of the bulk density in relation to the thermal conductivity coefficient λ of the GFG modified products is described by the quadratic function defined by the formula:

(4) $$\lambda_{1P}=0.55-0.017\;\cdot \;x_{1P}-0.0002\;\cdot \;x_{1P}{}^{2}\pm 0.02614$$

The indication in the formula: λ_1P_—thermal conductivity coefficient of a sand-lime product modified with GFG, size 0.25–0.5 mm, ρ_1P_—volume density. 

The adopted model explains the variability of the bulk density in 96%.

The research shows that the average value of the thermal conductivity coefficient λ of the tested silicate products decreases with an increase of GFG amount in the sample weight, and thus also with a decrease in its volume density. In the samples containing 30% of granules, it is lower than the traditional product by nearly 53%.

The results obtained indicate a correlation with the results covered by the literature, where granulated foam glass was used in concrete cubes [35]. An increase in heat-insulating parameters along with a decrease in volumetric density was also demonstrated in studies on the lightweight aggregate in the form of glass aggregate in the concrete composites [33,36].

The physical characteristics of the samples indicate that the use of granulated foam glass contributes to the production of environmentally friendly construction materials. Their total weight is significantly reduced while high thermal insulation is still maintained.

## 4. Conclusions

The results of the research carried out are as follows:

- The use of a light filler of granulated foam glass (GFG) in sand-lime products will allow the obtaining of products with good thermal insulation properties, i.e., characterized by a significantly reduced value of the thermal conductivity coefficient compared to traditional products;
- The conducted research made it possible to adjust the appropriate mathematical models that characterize the impact of the amount of fillers on the functional properties of the modified products;
- An increased effective porosity of the sand-lime products in the range of the analyzed diameters has a positive effect on the value of the thermal conductivity coefficient of products modified with the filler used;
- Although the GFG filler itself, according to the manufacturer’s declared properties, has a low degree of water absorption, the sand-lime samples autoclaved with the GFG filler are characterized by water absorption that increases significantly;
- The results apply only to samples modified with GFG of a specific fraction, i.e., the dimensions of 0.25–0.5 mm. At this stage of the research it is not possible to state that the changes in water absorption along with the bulk density and the changes in the thermal conductivity coefficient along with the bulk density expressed by the formulas presented in this article will be applicable to the use of GFG of a different fraction. A separate article will be devoted to this topic after an appropriate amount of research has been carried out on materials containing other fractions;
- In terms of the possibility of using the presented modification in the industry, especially from the technological and constructional point of view, the authors assumed that the compressive strength of the products should not be less than 5 MPa. Taking into account the changes in the performance characteristics described in this publication and in the article [39], it is indicated that the strength requirements are met by the modified GFG sand-lime samples where the amount in the sample does not exceed 25%. Such samples are characterized by a thermal conductivity coefficient of not less than 0.24 W/(m·K), and therefore it is more than 50% lower than for traditional products.
- Considering the value of water absorption, the possible use of the modified GFG products will involve the use of waterproofing in places exposed to contact with water.

## Figures and Tables

**Table 1:** Characteristics of ground quicklime.

**CaO + MgO** **[%]**	**MgO** **[%]**	**CO_2_** **[%]**	**SO_3_** **[%]**	**t_60_** **[min]**	**t_max_** **[min]**	**T_60_** **[°C]**
94.72	0.97	1.47	0.18	00:59	7:20	74.80
**Lime Active** **[%]**	**Capability** **[dm^3^/10 kg]**		**Passed through a Sieve**			
			**2 mm [%]**	**0.2 mm [%]**	**0.09 mm [%]**	
91.22	28.00		100.00	99.72	96.68	

**Table 2:** Physical parameters of the pore space—quantitative assessment.

Sample	Apparent Density, ρ [kg/m^3^]	Effective Porosity, P_e_ [%]	Average Diameter, d_50_ [μm]	Share of Mesopores, [%]	Share of Macropores, [%]
WP	1728	8.72	24	0	100
1P5	1575	35.49	20	22.9	77.1
1P10	1369	43.01	33	11.5	88.5
1P30	842	62.31	31	11.3	88.7

**Table 3:** Test results for the thermal conductivity coefficient and volume density of GFG modified lime-sand products with a diameter of 0.25–0.5 mm.

Sample	Thermal Conductivity Coefficient, λ [W/(m·K)]	Average Value of the Thermal Conductivity Coefficient, λ [W/(m·K)]	Volume Density, ρ [kg/m^3^]
WP	0.527	0.528	1679.1
	0.529		
1P5	0.502	0.505	1598.9
	0.507		
1P10	0.406	0.407	1451.3
	0.407		
1P20	0.272	0.274	1210.1
	0.276		
1P30	0.244	0.249	802.0
	0.254		

## Data Availability

Not applicable.

## References

[1] Kuśnierz A. (2010). Glass Recykling. Pract. Inst. Ceram. I Mater. Bud..

[2] Yang S., Lu J.X., Poon C.S. (2021). Recycling of waste glass in cement mortars: Mechanical properties under high temperature loading. Resour. Conserv. Recycl..

[3] Kaza S., Yao L.C., Bhada-Tata P., Van Woerdan F. (2018). What a Waste 2.0. Aglobal Snapshot of Solid Waste Management to 2050. Urban Development.

[4] Eurostat (European Commission) (2020). Energy, Transport and Environment Statistics, Statistical Book.

[5] Mallum I., Mohd.Sam A.R., Lim N.H.A.S., Omolayo N. (2021). Sustainable Utilization of Waste Glass in Concrete: A Review. Silicon.

[6] Chen Z., Poon C.S., Li J.S. (2020). Utilization of glass cullet to enhance the performance of recycled aggregate unbound sub-base. J. Clean. Prod..

[7] Kurpińska M., Grzyl B., Pszczoła M., Kristowski A. (2019). The Application of Granulated Expanded Glass Aggregate with Cement Grout as an Alternative Solution for Sub-Grade and Frost-Protection Sub-Base Layer in Road Construction. Materials.

[8] Lakov L., Jivov B., Ivanova Y., Yordanov S., Toncheva K. (2021). Alternative possibilities for application of foamed silicate materials. Mach. Technol. Mater..

[9] Omoding N., Cunningham L.S., Lane-Serff G.F. (2020). Effect of using recycled waste glass coarse aggregates on the hydrodynamic abrasion resistance of concreto. Constr. Build. Mater..

[10] Bostanci S.C. (2019). Engineering Properties and Sustainability Assessment of Recycled Glass Sand Concrete. Eur. J. Sci. Technol..

[11] Pingping H., Zhang B., Lu J.X., Poon C.S. (2020). ASR expansion of alkali-activated cement glass aggregate mortars. Constr. Build. Mater..

[12] Duraman S.B., Li Q. (2021). Recycled Glass Cullet as Fine Aggregate and Partial Cement Replacement in Concrete. IOP Conference Series: Materials Science and Engineering.

[13] Małek M., Łasica W., Jackowski M., Kadela M. (2020). Effect of Waste Glass Addition as a Replacement for Fine Aggregate on Properties of Mortar. Materials.

[14] Sikora P., Horszczaruk E., Skoczylas K., Rucinska T. (2017). Thermal properties of cement mortars containing waste glass aggregate and nanosilica. Procedia Eng..

[15] Bostanci S.C. (2020). Use of waste marble dust and recycled glass for sustainable concrete production. J. Od Clean. Prod..

[16] Dachowski R., Kostrzewa P. (2016). The use of waste materials in the construction industry. Procedia Eng..

[17] Owsiak Z., Kostrzewa P. (2017). Efects of Bentonite Additives on Autoclaved Sand-Lime Product Properties. IOP Conference Series: Materials Science and Engineering.

[18] Dachowski R., Stępień A. (2014). Impact of modification of sand-lime mass with organic compounds on the microstructure and mechanical features of silicate bricks. Environmental Engineering. Proceedings of the International Conference on Environmental Engineering.

[19] Stępień A., Kostrzewa P., Dachowski R. (2019). Influence of barium and lithium compounds on silica autoclaved materials properties and on the microstructure. J. Clean. Prod..

[20] Stepien A., Potrzeszcz-Sut B., Kostrzewa P. (2018). Influence and application of glass cullet in autoclaved materials. IOP Conference Series: Materials Science and Engineering.

[21] Jasińska I. (2013). The properties of silicate products modified by LDPE granulates. Struct. Environ..

[22] Kostrzewa P., Stępień A. (2017). Autoclaved Sand-Lime Products with a Polypropylene Mesh. IOP Conf. Ser. Mater. Sci. Eng..

[23] Dachowski R., Nowek M. (2016). Landfill leachate as an additive in sand-lime products. Procedia Eng..

[24] Fang Y., Gu Y., Kang Q., Wen Q., Dai P. (2011). Utilization of copper tailing for autoclaved sand–lime brick. Constr. Build. Mater..

[25] Stepien A., Potrzeszcz-Sut B., Prentice D.P., Oey T.J., Balonis M. (2020). The Role of Glass Compounds in Autoclaved Bricks. Buildings.

[26] Borek K., Czapik P., Dachowski R. (2020). Recycled Glass as a Substitute for Quartz Sand in Silicate Products. Materials.

[27] Stepień A., Leśniak M., Sitarz M. (2019). A Sustainable Autoclaved Material Made of Glass Sand. Buildings.

[28] Stępień A. (2021). Analysis of Porous Structure in Autoclaved Materials Modified by Glass Sand. Crystals.

[29] Schumacher K., Saßmannshausen N., Pritzel C., Trettin T. (2020). Lightweight aggregate concrete with an open structure and a porous matrix with an improved ratio of compressive strength to dry density. Constr. Build. Mater..

[30] Šeputyte-Jucike J., Sinica M. (2016). The effect of expanded glass and polystyrene waste on the properties of lightweight aggregate concreto. Eng. Struct. Technol..

[31] Chung S.-Y., Abd Elrahman M., Sikora P., Rucinska T., Horszczaruk E., Stephan D. (2017). Evaluation of the Effects of Crushed and Expanded Waste Glass Aggregates on the Material Properties of Lightweight Concrete Using Image-Based Approaches. Materials.

[32] Limbachiya M., Meddah M.S., Fotiadou S. (2012). Performance of granulated foam glass concrete. Constr. Build. Mater..

[33] Vaganov V., Popov M., Korjakins A., Šahmenko G. (2017). Effect of CNT on Microstructure and Mineralogical Composition of Lightweight Concrete with Granulated Foam Glass. Procedia Eng..

[34] Bubenik J., Zach J. (2019). The use of foam glass based aggregates for the production of ultra-lightweight porous concrete for the production of noise barrier wall panels. Transp. Res. Procedia.

[35] Asztalos F., Kocserha I. (2020). Laminar foam glass as a lightweight concrete aggregate. J. Phys. Conf. Ser..

[36] Kurpińska M., Ferenc T. (2020). Experimental and Numerical Investigation of Mechanical Properties of Lightweight Concretes (LWCs) with Various Aggregates. Materials.

[37] Dachowski R., Jasinska I. (2012). The effect of adding foamed glass granules on porosity of silicate products. Arch. Inst. Civ. Eng..

[38] Jasińska I. (2014). Effect of modification of sand-lime products on their basic functional properties. Tech. Trans..

[39] Jasińska I. (2019). Effect of foam glass granule fillers modification of lime-sand products on their microstructure. Open Eng..

[40] (2015). PN-EN 772-1+A1:2015-10 Methods of Test for Masonry Units—Part 1: Determination of Compressive Strength.

[41] (2018). PN-EN ISO 14688-2:2008-05 Geotechnical Identification and Research—Soil Determination and Classification—Part 2: Classification Rules.

[42] (2001). PN-EN 772-13:2001 Masonry Test Methods—Part 13: Determination of the Net and Gross Density of Masonry Members in the Dry State (Excluding Natural Stone).

[43] (2017). PN-EN 771-2+A1:2015-10 Requirements for Masonry Elements. Part 2: Calcium Silicate Masonry Elements.

[44] (2011). PN-EN 772-21:2011 Methods of Test for Masonry Units—Part 21: Determination of Water Absorption of Clay and Calcium Silicate Masonry Units by Cold Water Absorption.

[45] Petri M., Jan Małolepszy E. (2013). Podstawy Technologii Materiałów Budowlanych i Metody Badan.

[46] Semyrka R., Jarzyna J.A., Krakowska P.I., Semyrka G. (2015). Zastosowanie analizy porozymetrycznej dla oceny przestrzeni porowej skał w profilach utworów karbonu dolnego i karbonu środkowego północno-zachodniej Polski. Gospod. Surowcami Miner..

[47] Semyrka R., Semyrka G., Zych I. (2008). Zmienność parametrów petrograficznych subfacji dolomitu głownego zachodniej strefy półwyspu grotowa w świetle badań porozymetrycznych, Zeszyty Naukowe AGH. Geologia.

[48] Zapotoczna-Sytek G., Mamont-Cieśla K., Rybarczyk T. (2012). Naturalna promieniotwórczość wyrobów budowlanych, w tym autoklawizowanego betonu komórkowego. Przegląd Budowlany.

[49] Schober G. Porosity in autoclave aerated concrete (AAC): A review on pore structure, types of porosity, measurement methods and effects of porosity on properties. Proceedings of the 5th International Conference on Autoclaved Aerated Concrete.

[50] Firkowicz-Pogorzelska K. (2011). Metodyka określania wartości obliczeniowej współczynnika przewodzenia ciepła materiałów budowlanych. Prace Inst. Tech. Bud..

[51] International Organization for Standardization (ISO) (1991). ISO 8301:1991 Thermal Insulation—Determination of Steady-State Thermal Resistance and Related Properties—Heat Flow Meter Apparatus.

